# Supplementary material for: Inhibitory Effects of 3-Methylcholanthrene Exposure on Porcine Oocyte Maturation
Source: Int J Mol Sci. 2023 Mar 14;24(6):5567. doi: 10.3390/ijms24065567 (PMC10058619; doi:10.3390/ijms24065567)
Supplement: Supplementary file 1 [file ijms-24-05567-s001.zip › ijms-2267902-supplementary.pdf]

Notes for each gene:

Table S1. Porcine-specific primer sequences used in this study

Gene symbol	Primer sequences (5'-3')	Product size (bp)	GenBank accession number
BCL2	F: GGTACCGGAGGGCATTCAGT<br>R: TCCCGGAAGAGTTCGTTCAC	100	NC_010443.5
BAX	F: TGCTTCAGGGTTTCATCC<br>R: AGACACTCGCTCAACTTC	112	NC_010448.4
CASPASE3	F: GGATTGAGACGGACAGTG<br>R: CGCCAGGAATAGTAACCAG	109	NM_214131.1
TNFNIP6	F: TCATAACTCCATATGGCTTGAAC<br>R: TCTTCGTACTCATTTGGGAAGCC	396	NM_001159607.1
PTX3	F: TCAGTGCCTGCATTTGGGTC<br>R: CTACATGCCCTTGTTCAGAA	225	GQ_412351
PIGS2	F: ATGATCTACCCGCCTCACAC<br>R: GCAGCTCTGGGTCAAACTTC	279	AF_207824
HAS2	F: GAAGTCATGGGCAGGGACAATTC<br>R: TGGCAGGCCCTTTCTATGTTA	407	NM_214053
CD44	F: GGATGTGGTCCTGGTTTGGT<br>R: GGTTTCGTGCCTCTTGGTTG	122	XM_013994425.2
EF1α1	F: ATTGTTGCTGCTGGTGTTG<br>R: TCATATCTCTTCTGGCTGTAGG	161	NM_001097418-2

F: forward,
R: reverse

BCL2 is an apoptosis suppressor gene.
BAX and *CASPASE3* are pro-apoptotic genes.
EF1 α 1 is an internal reference gene.

Table S2. List of primary and secondary antibodies used in this study

Primary antibody	Species	Vendor	Cat.no. and dilution
F-actin	Mouse	EMD Millipore Corp USA	MAB15010 (1:200)
α -Tubulin	Mouse	Sigma	F2168 (1:200)
γ H2A.X	Mouse	abcam	Ab26350 (1:200)
AC-Tubulin	Mouse	Sigma	T7451 (1:100)
PNA-FITC		Sigma-Aldrich	L7381 (1:10)
**Secondary antibody**	**Species**	**Vendor**	**Cat.no. and dilution**
Alexa Fluor 488 anti-mouse IgG	Goat	Invitrogen	A11029 (1:200)
